# Community-based interventions to improve adolescent nutrition: a scoping review

**DOI:** 10.1186/s12889-025-25617-5

**Published:** 2025-12-30

**Authors:** Dewi Nurlaela Sari, Dany Hilmanto, Deni K. Sunjaya, Puspa Sari

**Affiliations:** 1 Doctoral Study Program, Faculty of Medicine, Universitas Padjadjaran, Bandung, Jawa Barat 45363 Indonesia; 2 Department of Child Health, Faculty of Medicine, Hasan Sadikin Hospital, Universitas Padjadjaran, Bandung, Jawa Barat 45363 Indonesia; 3 Department of Public Health, Faculty of Medicine, Universitas Padjadjaran, Bandung, Jawa Barat 45363 Indonesia

**Keywords:** Adolescent nutrition, Community-based interventions, Healthy eating behaviors, School-based nutrition programs

## Abstract

**Background:**

Adolescents are an age group that is vulnerable to nutritional problems due to changes in eating patterns influenced by social, economic, and environmental factors. The trend of consuming fast food, sugary drinks, and low intake of fruits and vegetables has led to an increase in the prevalence of obesity and non-communicable diseases among adolescents. Various community-based interventions have been developed to address this problem by involving schools, families, and social environments as part of a behavior change strategy. This approach is considered more effective because it takes into account various factors that influence adolescent eating habits holistically.

**Objective:**

To explore various community-based interventions to improve adolescents nutrition.

**Method:**

This scoping review was conducted by searching articles from three major databases, namely CINAHL, PubMed, and Scopus. Keywords used in the search included “adolescent nutrition,” “community-based interventions,” “school-based nutrition programs,” and “healthy eating behaviors.” The inclusion criteria applied included original research articles written in English, published in the period 2015–2025, and involving adolescent samples in the interventions studied. Data from articles that met the criteria were extracted using manual tables and analyzed descriptively qualitatively to identify patterns of interventions and factors influencing their effectiveness.

**Results:**

From 1,079 records, 12 studies met inclusion criteria (8 randomized/cluster-randomized trials, 2 quasi-experimental, 1 pre–post, 1 longitudinal survey). Four intervention categories emerged: school-based education, school food environment modifications, digital tools, and theory-driven behavior change (HBM/SCT/CBT). Multicomponent programs more consistently improved diet quality (e.g., higher fruit–vegetable intake; lower sugar-sweetened beverages) than single-component strategies; in longer follow-up, a reduction in waist circumference, but not BMI was observed. Comparative synthesis indicated that multicomponent programs integrating education, healthier food provisioning, and family or community engagement produced more consistent improvements in diet than single-component strategies. Digital tools were most effective when coupled with self-monitoring and caregiver involvement, while environment-only changes showed limited impact on intake and adiposity over short follow-up.

**Conclusion:**

Community-based strategies are most effective when delivered as integrated packages that align education with supportive food environments and structured caregiver engagement, ideally over ≥ 6–12 months with monitoring/feedback. Selecting sensitive outcomes (e.g., waist circumference, diet quality indices) clarifies early effects, whereas BMI may remain unchanged over shorter horizons.

## Introduction

Nutritional problems in adolescents have become a global concern because of their significant impact on individual and community health [1]. Global trends show that adolescents experience multiple nutritional challenges, including malnutrition, micronutrient deficiencies, and increasing prevalence of obesity due to unhealthy diets and sedentary lifestyles [2]. Inadequate nutrition can cause growth disorders, decreased immunity, and decreased concentration and academic achievement [3]. Meanwhile, in the long term, poor nutritional status contributes to an increased risk of non-communicable diseases, such as diabetes mellitus, hypertension, and cardiovascular disease in adulthood [4]. Adolescence is a critical phase in life marked by rapid physical growth, significant cognitive development, and complex emotional changes. During this period, nutritional needs increase substantially to support bone growth, muscle development, and maturation of the reproductive system [5].

The prevalence of malnutrition in adolescents continues to increase globally, reflecting the complex health challenges faced by countries. According to the World Health Organization (WHO), around 21% of adolescents are obese or overweight, while millions more suffer from malnutrition, especially in low- and middle-income countries [6]. Data from UNICEF also shows that around 45 million children and adolescents experience wasting (acute thinness), while 149 million others experience stunting (stunted growth) due to chronic nutritional deficiencies [7]. The phenomenon of the double burden of malnutrition, namely the existence of undernutrition and obesity in one population, further complicates nutritional prevention and intervention efforts [8].

Adolescence is therefore a critical window for targeted public health action, as dietary behaviors established during this period often persist into adulthood [9]. While individual-level and clinical interventions can address specific nutritional deficiencies, they may have limited reach and sustainability, especially in low-resource settings [10]. In contrast, community-based approaches address not only the biological and individual determinants of nutrition but also the social, environmental, and cultural factors that shape adolescents’ eating behaviors. By engaging multiple stakeholders, such as families, schools, local governments, and community organizations, these interventions can create supportive environments that reinforce healthy dietary habits, making them a strategic priority in improving adolescent nutritional health [11].

Community-based approaches to nutrition interventions are becoming increasingly important given the limitations of individual and clinical approaches in improving adolescent nutritional status broadly. Interventions that focus only on individuals often fail to reach larger groups and be sustainable, especially in communities with limited access to health services [12]. In contrast, a community-based approach offers a more holistic solution by engaging with the various social, environmental, and cultural factors that influence adolescents’ eating habits and lifestyles [13]. The basic principles of community-based interventions include the active participation of various stakeholders, including families, schools, health workers, community organizations, and governments, who work together to create an environment that supports healthy eating patterns and better nutritional behaviors [14].

Various forms of community-based interventions have been developed to improve adolescent nutritional health with a more inclusive and sustainable approach. One of the main strategies is school-based and community-based nutrition education programs, which aim to increase adolescent knowledge and awareness of healthy eating patterns and the importance of balanced nutritional consumption [15, 16]. These programs often involve nutrition education curricula, interactive workshops, and parent and teacher involvement in shaping better eating habits. In addition, digital and social media-based health promotion campaigns are increasingly being used to reach adolescents more effectively, given their high use of technology [17]. On the other hand, community-based interventions also include initiatives to increase access to healthy food through local policies, such as nutritious food subsidy programs, the provision of healthy canteens in schools, and the development of local food markets that sell healthy products at affordable prices [16]. This diverse approach shows that community-based interventions should not only focus on education, but also on environmental changes that support healthy eating patterns for adolescents [3].

Although a variety of community-based nutrition interventions have been implemented, there are still research gaps that limit understanding of the effectiveness and sustainability of these programs. One major limitation is the lack of studies that systematically review different forms of community-based interventions, making it difficult to identify the most effective models for improving adolescent nutritional status [13]. In addition, data on success factors and challenges in program implementation are still limited, especially related to aspects of sustainability, community involvement, and long-term effectiveness [18, 19]. This limitation is compounded by the lack of research addressing adaptation of interventions across cultural and social contexts, given that each community has unique characteristics that influence program acceptability and success. Therefore, further research is needed that not only evaluates the impact of interventions but also examines adaptation strategies that may enhance program effectiveness across settings. This scoping review aims to explore community-based interventions to improve adolescent nutritional health.

## Methods

### Study design

This study used a scoping review design following the Arksey and O’Malley framework, complemented by Levac’s refinement and the Joanna Briggs Institute (JBI) guidance. This approach was chosen because it allows for a broad exploration of the available literature, identifies research gaps, and provides a comprehensive understanding of the trends in interventions that have been implemented [20]. Scoping reviews are also more flexible than systematic reviews, allowing the synthesis of diverse intervention approaches without the need for strict quantitative meta-analysis. The stages in this scoping review included five steps: (1) identifying research questions based on the PCC (Population, Concept, Context) framework; (2) identifying relevant studies through systematic searches in academic databases; (3) selecting articles based on predetermined inclusion and exclusion criteria; (4) extracting and charting key information from each identified article; and (5) analyzing and reporting results thematically to understand intervention patterns and research gaps. The database searches were conducted in January 2025.

### Search strategy and eligibility criteria

A systematic literature search was conducted in three major databases, namely Scopus, PubMed, and CINAHL, which were selected because of their broad coverage in the fields of public health, nutrition, and nursing. We selected Scopus for its broad coverage of leading scientific journals. PubMed was chosen for access to evidence-based medical and health research. CINAHL was included because of its focus on community health and nursing. Articles were searched using a combination of Boolean operators and MeSH terms, such as “Adolescents” AND “Community-based interventions” AND “Nutritional health”, and their variations such as “Youth” OR “Teenagers”, and “Malnutrition” OR “Healthy eating promotion”. Each database has a specific search strategy that is tailored to the search features available. The PubMed search strategy is presented in full below to ensure reproducibility:

((“Adolescent“[MeSH Terms] OR adolescent* OR youth OR teenagers) AND (“Community-Based Participatory Research“[MeSH Terms] OR “community-based intervention*” OR “school-based intervention*” OR “community health services“[MeSH Terms]) AND (“Nutrition“[MeSH Terms] OR “Nutritional Status“[MeSH Terms] OR “Malnutrition“[MeSH Terms] OR “healthy eating” OR “dietary habit*”)) AND (english[lang])

**Fig. 1:**
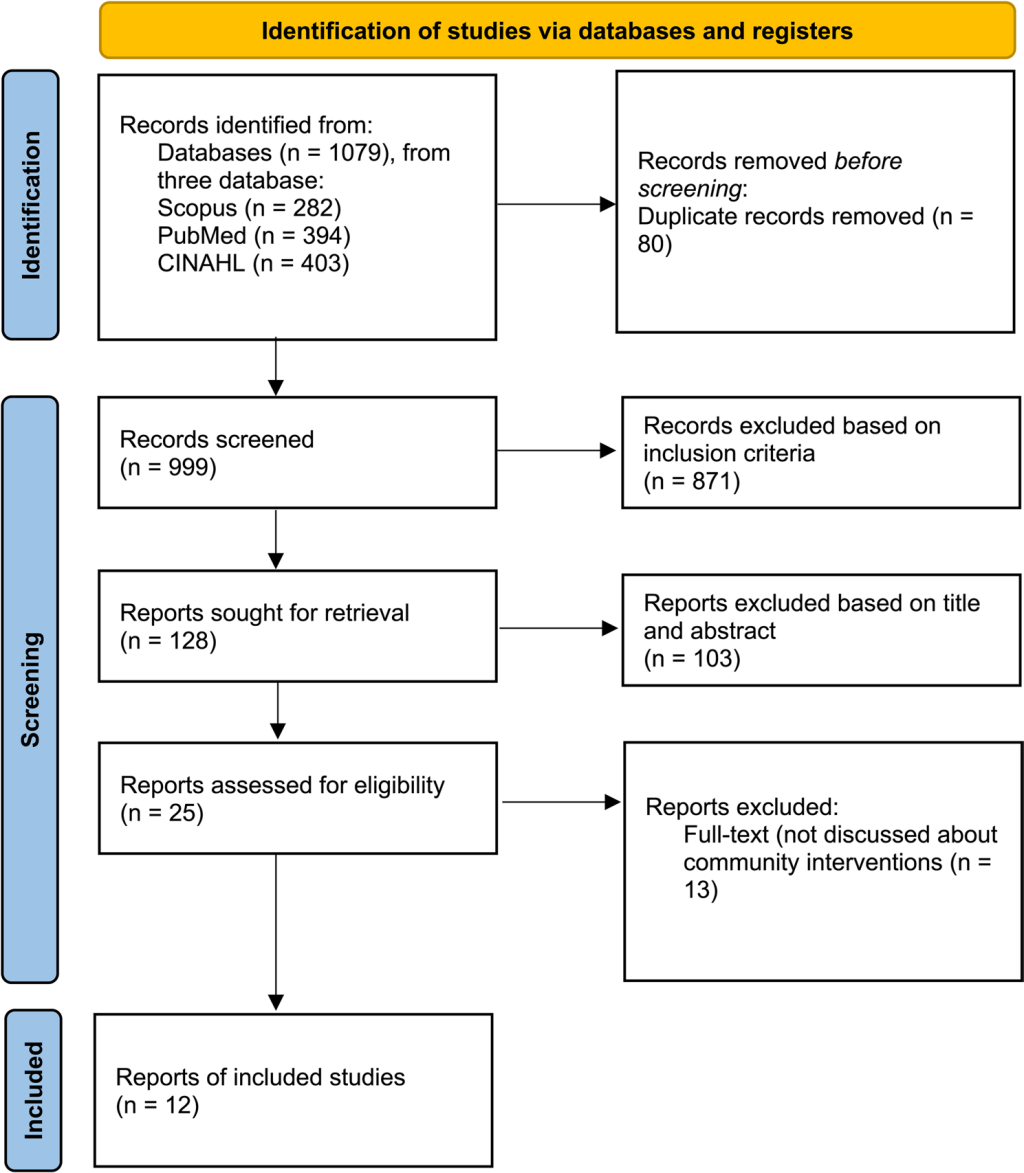
PRISMA flow diagram

For Scopus and CINAHL, search terms were adapted to database-specific subject headings and syntax. Boolean operators, truncations, and MeSH/subject headings were used to maximize retrieval. The search and selection process followed the PRISMA flow diagram for scoping reviews (Fig. 1).

### Inclusion and exclusion criteria

The inclusion criteria in this study were determined based on the PCC (Population, Concept, Context) framework, where the population (P) studied was adolescents aged 10–19 years, the concept (C) included community-based interventions aimed at improving nutritional health, and the context (C) included various community environments, such as schools, community health centers, and digital-based programs involving the community. Based on the PCC framework, the guiding research question for this scoping review is: “What types of community-based interventions have been implemented to improve nutritional health among adolescents aged 10–19 years, and what outcomes have been reported?”

The search was conducted in January 2025; inclusion years were limited to 2015–2025, English-language, peer-reviewed original studies. Studies were excluded if they lacked relevant outcome measures related to adolescent nutritional health, did not include participants within the 10–19-year age range, or were conducted outside community-based contexts. Literature reviews, editorials, commentaries, policy briefs without primary data, and studies without accessible full texts were also excluded. Grey literature and non-indexed sources were not included, as the review was limited to peer-reviewed articles from indexed databases to ensure methodological rigor and quality of evidence.

### Data extraction

Data extraction was performed manually using a table containing key information from each identified study, namely author name, study objectives, study design, sample characteristics, country of study, type of intervention applied, and main results of the study. Two independent authors with expertise in public health and community nutrition were responsible for the data extraction process to increase objectivity and accuracy. If there were differences of opinion in data extraction, discussions were held to reach a consensus. If no agreement was reached, a third researcher was involved as an arbitrator to determine the final decision. This strategy aims to minimize bias and ensure that the data collected is accurate and can be used in further analysis.

### Quality appraisal

Although quality appraisal is not mandatory for scoping reviews, it was conducted in this study to provide additional context on the methodological robustness of the included evidence. The appraisal followed the Joanna Briggs Institute (JBI) Critical Appraisal Checklists, with different instruments applied according to study design: 13 items for randomized controlled trials (RCTs) and 9 items for quasi-experimental studies [21]. The process was carried out independently by two authors. Any disagreements were resolved through discussion; if consensus was not reached, a fifth author was consulted to provide an additional assessment. Each checklist item was answered with “Yes,” “No,” “Unclear,” or “Not applicable.” Responses of “Yes” were assigned a score of 1, while “No,” “Unclear,” and “Not applicable” were assigned a score of 0.

Because this is a scoping review, certain studies with designs such as health surveys or pre–post studies could not be fully appraised with the standard JBI checklists. For these studies, appraisal was marked as Not Applicable (N/A) in Table 1. In addition, some checklist items were not relevant (NA) to particular study designs, resulting in different maximum possible scores (e.g., 8/9, 11/13, 12/13). The appraisal results were not used as exclusion criteria but were considered in the interpretation of the findings.

**Table 1:** JBI critical appraisal tool

Authors, Published Year	JBI Critical Appraisal Tool	Study Design
(Duus et al., 2022) [22]	92,3% (12/13)	RCT
(Ofosu et al., 2018) [27]	N/A	Health survey
(Fonseca et al., 2019) [23]	84,6% (11/13)	RCT
(Raikar et al., 2020) [24]	77,8% (7/9)	Pre-post study
(Moitra et al., 2021) [31]	92,3% (12/13)	RCT
(Ooi et al., 2021) [25]	84,6% (11/13)	RCT
(He et al., 2022) [29]	84,6% (11/13)	RCT
(Teo et al., 2021) [26]	88,9% (8/9)	Quasi-experimental
(Keshani et al., 2019) [30]	76,9% (10/13)	RCT
(Ghasab Shirazi et al., 2019) [32]	84,6% (11/13)	RCT
(Ardic & Erdogan, 2017) [32]	88,9% (8/9)	Quasi-experimental
(Ochoa-Avilés et al., 2017) [28]	92,3% (12/13)	RCT

N/A indicates domains that are not applicable to this study design or items that could not be appraised due to insufficient reporting. This label does not imply a high risk of bias.

Percentages are calculated as items met divided by items applicable. Checklists used: JBI RCT 13 items, JBI quasi experimental 9 items, JBI pre post studies treated under the JBI quasi experimental checklist with 9 items.

### Data analysis

Data analysis was conducted using a qualitative descriptive approach using thematic analysis methods to identify patterns and trends in community-based interventions that have been implemented in improving adolescent nutritional health. This thematic analysis approach is consistent with the recommendations of Arksey and O’Malley [20] and further refined by Levac et al., who emphasize the value of thematic synthesis in scoping reviews for mapping key concepts, identifying research gaps, and informing future research directions. The analysis process includes five main stages: (1) familiarization with the data, namely re-reading and understanding the extraction results to get an initial picture; (2) identification of initial codes, where data is coded based on the interventions carried out and the results obtained; (3) grouping codes into main themes, where codes that have similarities are categorized into broader themes; (4) review of themes, namely re-evaluation of the themes that have been formed to ensure that the findings produced can clearly describe various intervention approaches; and (5) reporting the results of the analysis in the form of narrative descriptions that explain the main patterns found in the literature. Two independent authors conducted this analysis separately to ensure the accuracy and precision of the results. If there are differences in interpretation, a third author will be invited to conduct additional analysis to reach consensus. This strategy aims to increase the validity of the findings and ensure that the results obtained can provide meaningful insights into community-based interventions in improving adolescent nutritional health.

## Results

From 1,079 records identified in Scopus, PubMed, and CINAHL, 80 duplicates were removed. After title and abstract screening and full text assessment against the inclusion criteria, 12 studies were included. The study selection process is presented in the PRISMA ScR flow diagram (Fig. 1). 12 articles were obtained according to the discussion of community-based interventions to improve nutritional health in adolescents (Table 2).

**Table 2:** Data extraction

No	Author(s) & Year	Country	Purpose	Sample	Methods	Interventions	Duration	Outcomes & Measures	Key Findings
1	(Duus et al., 2022) [22]	Denmark	Assessing the effectiveness of healthy school interventions on high school students’ meal frequency and eating habits.	4577 high school students	RCT Cluster	**The “Healthy High School” (HHS) multicomponent intervention**: consisting of a curriculum promoting healthy eating patterns, structural and organizational initiatives in schools, workshops, and smartphone applications to support healthy eating habits. Compared with a control group that carried out school habits as usual.	9 months	Meal frequency (days/week); water (servings/day); fruit & veg (servings/day; 1 ≈ 80 g)	There was no significant difference between the intervention and control groups.
2	(Ofosu et al., 2018) [27]	Canada	Assessing the long-term impact of Comprehensive School Health (CSH) on adolescent health	540 junior high and high school students	Health survey	**“APPLE Schools” Intervention**, a healthy school program that includes promoting healthy eating, increasing physical activity, and mental support for students. There is no explicit comparison group.	7 years	Nutrition knowledge (score 0–100); attitudes (scale); self-efficacy (scale); eating habits (servings/day); PA (min/week or days/week)	There is no significant difference between APPLE Schools graduates and other schools
3	(Fonseca et al., 2019) [23]	Brazil	Assessing the effectiveness of nutrition interventions using visual representations in improving healthy eating knowledge & practices	461 adolescents (273 intervention, 188 control)	RCT	**Nutrition education intervention based on visual approach**, consisting of 3 meeting sessions discussing the principles of healthy eating, food classification, reading food labels, and analyzing food advertisements using visual materials such as food pictures, food models, and food packaging replicas. Compared with a control group that did not receive the intervention.	Not mentioned	Knowledge (% correct); veg intake (servings/day; 1 ≈ 80 g); SSB (servings/day; 1 ≈ 355 mL)	The intervention group showed increased vegetable consumption & reduced soft drinks
4	(Raikar et al., 2020) [24]	India	Assessing the effectiveness of nutrition education sessions using flipcharts in adolescent girls	265 female students in grade 9	Pre-post study	**Nutrition education using flipcharts**, with material covering the nutritional needs of adolescent girls, the impact of malnutrition, and examples of healthy foods. Sessions were conducted in an interactive format, with food demonstrations and visual media. There was no control group.	Not reported	Knowledge (score %)	Significant increase in knowledge after intervention
5	(Moitra et al., 2021)	India	Assessing the effectiveness of Health Belief Model-based nutrition education in adolescents	498 students (292 intervention, 206 control)	RCT Cluster	**Nutrition education intervention based on Health Belief Model (HBM) theory**for 12 weeks. The program included weekly sessions for students and 3 educational sessions for parents. Behavioral communication strategies were used to increase awareness of the benefits of balanced nutrition and physical activity. Compared with a control group that did not receive the intervention.	12 weeks	Knowledge (score %); attitudes (scale); diet (servings/day; fruit/veg 1 ≈ 80 g; SSB 1 ≈ 355 mL); PA (min/week or days/week)	Significant improvement in knowledge, attitudes, & healthy behavior in the intervention group
6	(Ooi et al., 2021) [25]	Australia	Assessing the effectiveness of sugary drink interventions in schools	862 junior high school students	RCT	**School-based interventions to reduce sugary beverage consumption**, including changes to the school environment (limiting the availability of sugary drinks), nutrition education curriculum, and community and parent involvement in awareness campaigns. Compared with a control group with no changes.	6 months	SSB intake (servings/day; 1 ≈ 355 mL); energy (kcal/day); BMI (kg/m² or z)	There was no significant difference between the intervention and control groups.
7	(He et al., 2022) [29]	China	Assessing the effectiveness of salt reduction education applications on students and families.	Not mentioned	School-based RCT	**App-based educational intervention (“AppSalt”)**, which teaches children and families about salt reduction through a mobile app. Children are given homework to educate their families. Compared to a control group that did not receive this education.	12 months	Salt intake (g/day; urine/recall; Na→salt ×2.54); BP (mmHg)	Significant reduction in salt intake in the intervention group
8	(Teo et al., 2021) [26]	Malaysia	Assessing the effectiveness of school nutrition programs on children’s eating behavior & body composition	488 elementary school children	Quasi-experimental	**School Nutrition Program**, which included nutrition education for students, changes to the school cafeteria environment to provide healthy foods, and teacher and parent involvement. Compared to schools without intervention.	3 months	Knowledge (score %); healthy diet (servings/day); PA (min/week); BMI (kg/m² or z)	Increased nutritional knowledge & healthy eating behavior in the intervention group
9	(Keshani et al., 2019) [26]	Iran	Assessing the effectiveness of a Health Belief Model (HBM)-based nutrition education intervention in a collaborative learning context.	311 students (163 intervention, 148 control)	RCT	**HBM-based nutrition education intervention**, consisting of collaborative learning sessions over the course of an academic year. Materials covered the benefits of healthy eating, barriers to behavior change, and self-efficacy. Compared to a control group with no intervention.	1 academic year	Knowledge (%); risk perception (scale); self-efficacy (scale); diet quality (index/score)	Significant improvements in diet quality and behavioral factors in the intervention group
10	(Ghasab Shirazi et al., 2019) [30]	Iran	Evaluating the effectiveness of Social Cognitive Theory (SCT)-based interventions on adolescent girls’ dietary behavior.	230 female students (115 intervention, 115 control)	RCT	**SCT-based nutrition education intervention**, including a multicomponent educational package (for students, parents, and teachers), support groups, and participatory homework assignments. Compared with a control group that received only monthly lectures.	6 months	Breakfast frequency (days/week, 0–7); fruit & veg (servings/day; 1 ≈ 80 g); unhealthy foods (servings/day)	Increased consumption of healthy foods in the intervention group, but has not yet reached ideal status
11	(Ardic & Erdogan, 2017) [32]	Türkiye	Assessing the effectiveness of the COPE Healthy Lifestyles TEEN program in preventing obesity and improving mental well-being	87 junior high school students (45 intervention, 42 control)	Quasi-experimental	**COPE Healthy TEEN Program**, consisting of 15 healthy lifestyle education sessions based on Cognitive Behavioral Therapy (CBT), including strategies for building self-esteem, stress management, physical activity, and nutrition. Compared with a control group without intervention.	12 months	Fruit & veg (servings/day; 1 ≈ 80 g); PA (min/week); BMI (kg/m²); anxiety (scale)	Increased physical activity & stress management, decreased anxiety, but no significant difference in BMI vs. controls
12	(Ochoa-Avilés et al., 2017) [28]	Ecuador	Evaluating the impact of school interventions on adolescent dietary intake and waist circumference	1430 students (20 schools)	RCT Cluster	**Nutrition education program “ACTIVITAL”**, consisting of an educational toolkit, workshops for parents, and changes to the school cafeteria environment to provide healthy foods. Compared with a control group with no intervention.	28 months (2 stages)	Unhealthy foods (servings/day); sugar (servings/day or g/day; SSB 1 ≈ 355 mL); waist circ. (cm)	Decreased consumption of unhealthy foods & sugar, as well as decreased waist circumference in the intervention group

### Study characteristics

Among the 12 included studies, 8 were randomized or cluster randomized trials which is 66.7%, 2 were quasi experimental which is 16.7%, 1 was a pre post study without a control group which is 8.3%, and 1 was a longitudinal survey which is 8.3%. Interventions clustered into four categories. The first category was school based nutrition education which included visual and flipchart modalities. The second category was modifications to the school food environment. The third category was technology enabled or digital applications. The fourth category was theory driven behavior change including Health Belief Model, Social Cognitive Theory, and Cognitive Behavioral Therapy or COPE. Reported durations ranged from three to twenty eight months, and one study reported a seven year follow up. Summary characteristics are provided in Table 2.

#### Thematic synthesis of outcomes

##### Theme 1. Multicomponent programs show the most consistent dietary improvements

Across trials, multicomponent school anchored programs that combined classroom education with changes to canteen or cafeteria offerings and with parent or community engagement more consistently reported improvements in dietary behaviors than single component approaches. Reported outcomes included higher fruit and vegetable intake and lower consumption of sugar sweetened beverages [22–25]. In studies with longer follow up, reductions in waist circumference were observed, whereas body mass index generally showed no significant change within shorter to mid length follow up windows [23–26].

##### Theme 2. Environment only modifications show limited short term impact

Interventions focused solely on the school environment did not show consistent effects on total sugar sweetened beverage intake or body mass index when compared with controls over short follow up periods. For example, restricting availability of sugar sweetened beverages within schools did not reliably reduce overall consumption or adiposity markers relative to comparison groups [27].

##### Theme 3. Education is more effective when grounded in behavior change theory

Education oriented programs reliably improved nutrition knowledge, while effects on dietary practices were mixed. Studies that explicitly applied behavior change frameworks such as Health Belief Model, Social Cognitive Theory, or Cognitive Behavioral Therapy including COPE more frequently reported improvements in attitudes, self efficacy, breakfast frequency, and fruit and vegetable intake than information only education programs [26, 28–32].

##### Theme 4. Digital tools are effective when paired with self monitoring, feedback, and caregiver engagement

Digital applications were associated with clearer changes when they incorporated self monitoring, regular feedback, and caregiver involvement. A school anchored application that targeted household salt reduction reported significant reductions in measured salt intake compared with controls over a twelve month period [33]. School based technology supports without reinforcement components showed mixed adherence and mixed outcomes.

##### Theme 5. Dose, duration, and outcome sensitivity influence observability of change

Short and single session exposures were primarily associated with gains in knowledge and self efficacy. Programs delivered for at least six to twelve months more often reported broader changes in diet. Waist circumference was more likely than body mass index to change within the study horizons examined in this review [23–26].

## Discussion

This scoping review mapped community based interventions for adolescent nutrition and identified five consistent patterns. Multicomponent school anchored programs produced the most reliable dietary improvements, including better diet quality and lower central adiposity in longer follow up [34]. Similar advantages were reported in another school based program with extended exposure [24]. Education was more effective when grounded in behavior change theory, with trials showing improvements in attitudes and self efficacy alongside select dietary behaviors [30]. A separate study using a theory informed design reported gains in breakfast frequency and fruit and vegetable intake [35]. Environment only policies showed limited short term impact on total intake and adiposity in a cluster trial that restricted sugar sweetened beverages within schools [27]. Digital tools performed best when paired with self monitoring, feedback, and caregiver engagement, as demonstrated in a school anchored salt reduction program [33]. Program dose, duration, and outcome sensitivity influenced whether change was observed, with waist circumference responding earlier than body mass index in longer interventions [25]. Another trial with a twelve month horizon reported behavioral gains without concurrent change in body mass index [26].

### Interpretation of principal findings

The advantage of multicomponent packages aligns with a capability, opportunity, and motivation pathway in which classroom education builds capability and healthier canteen provision expands opportunity [23]. Caregiver engagement likely strengthens motivation and helps sustain behavior beyond school hours [24]. Environment only modifications did not consistently alter total sugar sweetened beverage intake or body mass index, indicating that availability changes inside school were insufficient on their own [27].

Education oriented programs reliably improved knowledge, while changes in practice were more frequent when designs used explicit behavior change frameworks such as Health Belief Model or Social Cognitive Theory [30]. Cognitive behavioral elements, including goal setting and problem solving, were associated with psychosocial improvements that support maintenance of new habits [13, 36]. Digital interventions were most effective when the behavioral target was specific and measurable and when families participated, as seen in the salt reduction trial with twelve month effects [33].

Implementation quality likely moderated observed effects. Variability in fidelity, adherence, and potential contamination between school clusters can attenuate diet and anthropometry outcomes, especially when intraclass correlation is not accounted for or when process data are sparse [37]. Differences in local food availability and affordability outside school may also dilute environment based changes made inside school settings, which helps explain heterogeneous results across countries and communities [38, 39]. Broader lifestyle factors such as sleep have documented links with sugar sweetened beverage consumption and may interact with school policies in ways that constrain detectable change over short follow up periods [40]. Longer running programs with clearer implementation structures appeared to sustain behavior change more reliably than shorter efforts, consistent with reports from long term evaluations in other adolescent cohorts [41].

The pattern of stronger effects in theory informed designs is also consistent with the availability of validated measurement tools for key constructs. Health Belief Model instruments used in school health contexts can capture changes in perceived risk and benefit, thereby making mediators visible and actionable within trials [39]. Evidence from nutrition education grounded in similar constructs shows that targeted cognitive and motivational shifts can translate into improved dietary practices when delivery is structured and reinforced [42, 43]. Family–school approaches that integrate environmental and behavioral strategies are particularly well positioned to convert these psychosocial gains into routine intake changes in adolescents [44]. At the same time, findings should be interpreted within the population boundaries of this review, since clinical groups such as pregnant adolescents or youth in cancer treatment face distinct nutritional constraints that limit generalizability [45].

Risks of bias particular to school-based cluster designs deserve explicit management. Contamination across clusters is common when students or staff share materials, when activities mix across classes, or when vendors supply multiple schools. Blinding is difficult given visible allocation, increasing performance effects; assessor blinding for self-reported diet is infeasible and inconsistently applied for anthropometry [46]. Baseline imbalances in socioeconomic context or diet quality, failure to account for intraclass correlation in sample size and analysis, differential attrition by cluster, partial implementation, and Hawthorne effects can all bias estimates [44]. Future trials should pre-specify cluster allocation with concealment where feasible, power and analyze for clustering, separate delivery teams across arms, and audit fidelity and contamination using structured checklists [47–50].

Differences between waist circumference and body mass index likely reflect outcome sensitivity during adolescence. Central adiposity measures can respond within typical program windows, whereas body mass index may remain unchanged despite better diet quality [25]. Evidence of behavioral improvement without body mass index change over twelve months supports this interpretation [26].

### Implications for implementation and policy

Programs should align curriculum based nutrition education with a healthier school food environment and structured caregiver engagement, delivered with planned reinforcement and simple monitoring and feedback loops [23]. Extending delivery for at least six to twelve months increases the likelihood of observable dietary change [24]. Digital components should include narrow targets, self tracking, scheduled feedback, and explicit caregiver roles to support adherence and effect [33]. For evaluation, diet quality indices and waist circumference should be reported alongside body mass index or body mass index for age to capture early and sustained impact [25].

Policy actions should reflect a whole of school approach while acknowledging the limits of environment only change observed in the beverage restriction trial [27]. Complementary measures that address availability and affordability of healthier options outside school can reduce compensatory purchasing and improve the likelihood of sustained impact [24].

### Strengths and limitations of the evidence base

The included studies span diverse settings and designs and provide convergent signals on program features associated with change. At the same time, heterogeneity in components and measures limits direct comparison of effect sizes, and many studies relied on self reported intake. School based cluster designs are also vulnerable to contamination and benefit from clearer reporting of intraclass correlation and fidelity procedures. Incomplete adherence reporting further constrains inference about dose and engagement.

### Priorities for future research

Future studies should extend follow up beyond twelve months and embed economic evaluation and equity analysis to inform scale up. Process evaluation should be standardized with fidelity checks and contamination audits, particularly in cluster designs. Digital trials should report engagement trajectories including retention and adherence and should prespecify monitoring and feedback schedules. Mixed outcome sets that include diet quality indices, psychosocial mediators, waist circumference, and age appropriate anthropometry will better capture early and sustained impact.

## Conclusions

This scoping review indicates that adolescent nutrition is most likely to improve when community based strategies are delivered as integrated packages that align curriculum based education, a healthier school food environment, and structured caregiver engagement. Programs that apply explicit behavior change theory and that include simple self monitoring and feedback show more consistent gains in diet quality, with waist circumference responding earlier than body mass index within typical study windows. Short and single session exposures mainly shift knowledge, whereas delivery for at least six to twelve months is more often associated with broader dietary change. Policy and practice should therefore prioritize multicomponent designs with planned reinforcement and monitoring, and should evaluate impact using sensitive proximal outcomes alongside anthropometry. Future research should extend follow up, report adherence and fidelity in a standardized way, and embed economic and equity analyses to support scale up and sustained implementation.

## Data Availability

All data generated or analysed during this study are included in this published article.
